# The contribution of indigenous knowledge to disaster risk reduction activities in Zimbabwe: A big call to practitioners

**DOI:** 10.4102/jamba.v10i1.493

**Published:** 2018-03-26

**Authors:** Ernest Dube, Edson Munsaka

**Affiliations:** 1 Department of Development Studies, Midlands State University, Zimbabwe; 2 Faculty of Commerce, National University of Science and Technology, Zimbabwe

## Abstract

This article examined the contribution of indigenous knowledge to disaster risk reduction activities in Zimbabwe. The current discourse underrates the use of indigenous knowledge of communities by practitioners when dealing with disasters’, as the knowledge is often viewed as outdated and primitive. This study, which was conducted in 2016, sought to examine this problem through analysing the potential contribution of indigenous knowledge as a useful disaster risk reduction intervention. Tsholotsho district in Matabeleland, North province of Zimbabwe, which frequently experiences perennial devastating floods, was used as a case study. Interviews and researcher observations were used to gather data from 40 research participants. The findings were that communities understand weather patterns and could predict imminent flooding after studying trees and clouds, and the behaviours of certain animal species. Local communities also use available local resources to put structural measures in place as part of disaster risk reduction interventions. Despite this important potential, the study found that the indigenous knowledge of disaster risk reduction of the communities is often shunned by practitioners. The practitioners claim that indigenous knowledge lacks documentation, it is not found in all generational classes, it is contextualised to particular communities and the knowledge cannot be scientifically validated. The study concluded that both local communities and disaster risk reduction practitioners can benefit from the indigenous knowledge of communities. This research has the potential to benefit communities, policymakers and disaster risk reduction practitioners.

## Introduction and background

Proponents of indigenous knowledge systems, such as disaster risk reduction scholars, have often contested that the indigenous knowledge of local communities can contribute significantly towards saving human lives and property from the negative consequences of disasters (Hiwasaki, Luna & Syamsidik 2014; McAdoo et al. 2006). The scholars’ argument has been that local knowledge from the grassroots level should not be ignored by authorities as it can help communities to prevent, mitigate, prepare for and recover from the impact of disasters (Jones 2012). Local people have certain capacities that have evolved over centuries and this capacity and knowledge have been tested over time and proven to be sustainable and effective in both reducing disasters and managing hazards (Shaw et al. 2008). There is evidence suggesting that countries that have succeeded in managing disasters have also employed the indigenous knowledge of local communities affected by disasters as a strategy (Iloka 2016; Rahman, Sakurai & Munadi 2016; Sethi et al. 2011). As observed by Nyong, Adesina and Elasha (2007), indigenous knowledge is important as it has facilitated the survival of local populations in Africa’s Sahel region in the wake of climate change and variability. However, the extent to which indigenous knowledge has been usefully applied in disaster risk reduction in Zimbabwe has been understudied, probably because of ignorance regarding the potential value of this knowledge.

Zimbabwe has been overwhelmed by with disasters of different types and magnitude in the recent past (Madamombe 2004). Some parts of the country, such as Tsholotsho and Muzarabani districts, have been affected by floods and droughts, whilst Bulilima and Mangwe districts have been affected by veld fires (Dube 2015; Mudavanhu 2014). These disasters, especially floods, have been a threat to human life and property of communities living in flood-prone areas. Apart from employing modern knowledge and technology in managing disasters, practitioners are encouraged to put on lenses that would enable them to see the importance of embracing locally generated, indigenous knowledge of local communities in dealing with natural disasters.

According to Mutasa (2015), farmers of smallholdings in Zimbabwe have for centuries depended on indigenous knowledge for survival in dealing with droughts and other hazards. However, the full utilisation of indigenous knowledge has been under threat, as this knowledge is often marginalised by practitioners in favour of modern disaster risk reduction knowledge. Currently, there have been calls for an increased utilisation of the indigenous knowledge of the local communities – knowledge which local people have used for generations to mitigate hazards and reduce disasters in their communities (Gaillard & Mercer 2012; United Nations International Strategy for Disaster Reduction [UNISDR] 2005).

The main focus of this study was the use of indigenous knowledge systems in understanding and managing disasters. The study sought to understand how indigenous knowledge systems of local communities can be effectively used in the management of different types of disasters in Zimbabwe, with Tsholotsho district in the Matabeleland North province, in the south-western part of Zimbabwe, as a case study. The objectives of the study were to analyse the contribution of indigenous knowledge to flood disaster risk reduction interventions; to identify indigenous knowledge systems in dealing with flood disasters available to local communities in the district; to analyse how local communities in Tsholotsho district can benefit through using indigenous knowledge in flood disaster risk reduction; to discuss how practitioners can take advantage of local communities’ indigenous knowledge when dealing with flood disasters; and to analyse reasons why practitioners are sceptical of the indigenous knowledge of local communities in managing flood disasters.

## The context of the problem in Tsholotsho district

Communities living in Tsholotsho district have been experiencing disasters resulting from flooding. Although the district lies in Zimbabwe’s ecological region 5, which is usually a dry area characterised by low rainfall levels, many communities in the district are situated in low-lying areas, making them prone to perennial flooding. As such, the communities have experienced severe flooding in the past, resulting in the loss of life and destruction of property (Dube & Chiwanga 2014). As an intervention, the government of Zimbabwe and its cooperating partners, which include non-governmental organisations, humanitarian organisations and United Nations’ agencies, have been responding to the disaster needs following modern intervention regimes that focus on providing food aid but ignoring the indigenous knowledge in the prevention, mitigation, preparedness, response and recovery regime. It is the authors’ contention that the exclusion of the indigenous knowledge in managing flooding in Tsholotsho exacerbates the occurrence and effects of flooding in the flood-prone areas where human settlements are located close to the Gwayi, Zombani and Manzamnyama rivers and low-lying localities.

## Indigenous knowledge systems in disaster risk reduction

Indigenous knowledge systems have existed as part of human life from yesteryear and this practice is important as it has shaped how people interact with their environment. Dekens (2007) observes that local knowledge and practices to improve disaster risk reduction have grown since the 1970s. As such, this knowledge gained recognition and prominence in the 1990s in the field of disaster risk reduction and in issues associated with climate change (Hiwasaki, Luna & Syamsidik 2014). However, despite the recognition of the important role that indigenous knowledge plays in reducing the risk of disasters and adapting to climate change, this knowledge has not featured prominently in disaster policy and science (Adger et al. 2011). Some disaster risk reduction practitioners are still doubtful of its relevance and effectiveness, and regard indigenous knowledge as being closed, parochial, unintellectual, primitive and emotional (Herbert 2000; Mitchel 1995).

A lack of clarity of what constitutes indigenous knowledge has not helped its applicability. Various scholars have differing views of what indigenous knowledge entails (Fabiyi & Oloukoi 2013). According to Kelman et al. (2012), indigenous knowledge is regarded as local knowledge or traditional knowledge that is derived from local communities. Agrawal (1995) defines indigenous knowledge as knowledge that is passed down through generations, gained from knowledge of the environment which is revealed through intuition, dreams or visions. According to Melchias (2001), indigenous knowledge refers to what indigenous people know and do and what they have known and done for generations – these being practices that have evolved through trial and error and proved flexible enough to cope with change. Chianese (2016) concurs that indigenous knowledge is the knowledge and know-how accumulated across generations, tested and adapted through millennia, and which guide indigenous societies in their interactions with their surrounding environments. A closer look at all these concepts points to the fact that indigenous knowledge is locally grown, passed down from one generation to another and that the knowledge is gained over many years.

As far as the management of disasters is concerned, communities have also relied on their indigenous knowledge to minimise the impact of disasters. Those communities that have embraced indigenous knowledge have managed to save lives and property from various types of natural disasters. For instance, in the aftermath of the 2004 Indian Ocean earthquake and tsunami, indigenous knowledge helped communities to survive the disaster (Meyers & Watson 2008; Rungmanee & Cruz 2005). In some contexts, indigenous knowledge has been widely used to complement and expand scientific knowledge and to empower local communities (Makhanu et al. 2007). However, the Tsholotsho case is devoid of the inclusion of indigenous knowledge by disaster practitioners, despite the potential value the knowledge has in reducing the impact of flooding.

### The importance of indigenous knowledge systems to communities in disaster risk

Indigenous knowledge systems present many alternatives to governments, scientists, practitioners and local communities on how they should approach different disasters. Mwaura (2008) argues that indigenous knowledge can empower members of a community to take leading roles in activities aimed at reducing disaster risk. For instance, mixed cropping is a form of indigenous knowledge which can be applied to improve the yield of various crops, so that alternative crops are available for consumption if other crops fail (Mwaura 2008). A community that possesses vast indigenous knowledge of disaster risk reduction is able to take care of itself and also able to deal with disasters with minimum external support. Through the use of their indigenous knowledge, people can deal with different kinds of hazards and disasters before the arrival of disaster risk reduction practitioners.

According to Mutasa (2015), indigenous knowledge is very important in planning for community development. This shows that indigenous knowledge can be used as a planning tool by local communities. Such knowledge can be used to predict the occurrence of disasters and their impact so that proper interventions are adopted. Nyong et al. (2007) further note that developmental strategies cannot be successful without incorporating indigenous knowledge. In communities where indigenous knowledge is not widely used, such communities have continued to suffer severe consequences from natural disasters. Local people have a wealth of experience and understanding pertaining to their local environment. Therefore, they possess incorporating information that can be relied upon to help their communities plan for and better manage disasters in order to reduce the risk and impact. Their involvement in disaster risk reduction programmes is therefore important. It has been observed that disaster-affected people are not hopeless victims (Sphere Project 2011), but are citizens who possess certain capacities and important indigenous knowledge that practitioners can use.

Indigenous peoples knowledge provides information and insight that complement conventional science and environmental observations. It can also provide a holistic understanding of the environment, natural resources and culture, and the human interrelation between them (Galloway-McLean 2010; Nakashima et al. 2012; Tauli-Corpuz et al. 2009; Tebtebba Foundation 2009). Therefore, ignoring indigenous communities’ involvement in the planning stages of programmes affecting their lives would likely result in a negative project output and impact. Shaw et al. (2008) summarise the importance indigenous knowledge systems of local communities and also give reasons why such knowledge should be considered as part of policies for disaster risk reduction. Indigenous knowledge is important in that it can be transferred and adapted to other communities in similar situations; it encourages community participation and empowers communities in reducing disaster risk; it can provide invaluable information about the local context; and its non-formal means of dissemination can serve as a model for education about disaster risk reduction.

The value of indigenous knowledge is not limited to communities at risk. Instead, it also brings an invaluable contribution to the field of disaster risk reduction. As such, disaster risk reduction practitioners, whose knowledge has a bias towards modern technology (Maferetlhane 2012), can benefit immensely from the indigenous knowledge of communities. Mercer et al. (2010) argue that the indigenous knowledge of local communities is usually underappreciated by practitioners, as is regarded in some quarters as being inferior to scientific knowledge. However, the contribution of indigenous knowledge to practitioners’ activities is emphasised by Kallard (2000), who argues that indigenous knowledge has an advantage over Western science in that information is tested in the context of survival. Hence it is not just true or false in a dispassionate way but is effective in providing a means of survival. However, in order for practitioners to realise the greater benefits of indigenous knowledge, they need to integrate this with scientific knowledge when dealing with hazards and disasters so that the two types of knowledge can complement each other. A case in point is the Indian Ocean tsunami, which has been credited with sparking interest in indigenous knowledge and its integration with scientific knowledge for disaster risk reduction (Mallapaty 2012). Such a fusion of the two kinds of knowledge ensured a combination of their strengths, resulting in disaster risk reduction activities becoming more effective.

According to the UNISDR (2015), the Sendai Framework for Disaster Risk Reduction 2015–2030, adopted by the Third United Nations World Conference, advocates for the use of indigenous peoples’ knowledge and practices to complement scientific knowledge in disaster risk assessment. The framework recognises that indigenous peoples, through their experience and traditional knowledge, provide an important contribution to the development and implementation of plans and mechanisms, including early warning (UNISDR 2015). Therefore, indigenous knowledge is a vital component of disaster risk reduction.

### Limitations of indigenous knowledge for disaster risk reduction intervention

Although the indigenous knowledge of local communities is regarded as an important element for managing disasters, there are some limitations associated with it. One major problem with indigenous knowledge is that it is not wholly trusted by many in the communities, as well as disaster risk reduction practitioners. Scepticism by disaster risk reduction practitioners regarding the use of indigenous knowledge arises as a result of the fact that such knowledge lacks documentation. For instance, Banda (2008) asserts that indigenous knowledge systems are regarded as very rigid and non-documented, backward and superstitious. This claim might be motivated by Agrawal (1995), who defines indigenous knowledge as knowledge that is passed down from generation to generation, gained from knowledge of the environment which is revealed through intuition, dreams or visions. Anecdotal evidence also suggests that antagonists of indigenous knowledge further argue that it is not found in all generational classes, is contextualised to particular communities and cannot be scientifically validated (Matsui 2015).

However, proponents of indigenous knowledge, including academics, have different views pertaining to the factors that contribute to a lack of trust and belief in the indigenous knowledge of communities (Mawere 2010; Ngulube, Masuku & Sigauke 2011; Shizha 2006). Naidoo (2007) argues that the uses of indigenous means of survival have not always proved to be sustainable. This suggests that indigenous knowledge may not always be a right intervention for all hazards and disasters affecting human communities. According to Tanyanyiwa and Chikwanha (2011), indigenous knowledge is sometimes accepted uncritically because of naive notions that whatever indigenous people do is naturally in harmony with the environment. This negativity is especially construed as absolute truth when compared with modern science, where experts claim that its vast knowledge is universal; no one person, authority or social group would claim knowing it all (Sillitoe, Dixon & Barr 2005); and it has been scientifically proven to be correct. According to Grenier (1998), another limitation of indigenous knowledge is that sometimes the knowledge may be wrong or even harmful to people. This implies that at times practices based on indigenous knowledge may exacerbate a community’s vulnerability to disasters.

However, despite all its limitations, the paper argues that indigenous knowledge, if given space, would continue to play a significant role for local communities and practitioners in disaster risk reduction. If anything, local people should be enabled to actively participate in decision-making processes at regional, national and local levels (Pearce et al. 2009). Feldt (2011) also argues that for increased involvement, local people’s lifestyles and worldview must be respected through strengthening and recognising their rights over land, territories and resources. Their knowledge can provide important insights into the processes of observation, adaptation and mitigation of the consequences of climate change (Chianese 2016).

## Study site and the research methodology

**FIGURE 1:**
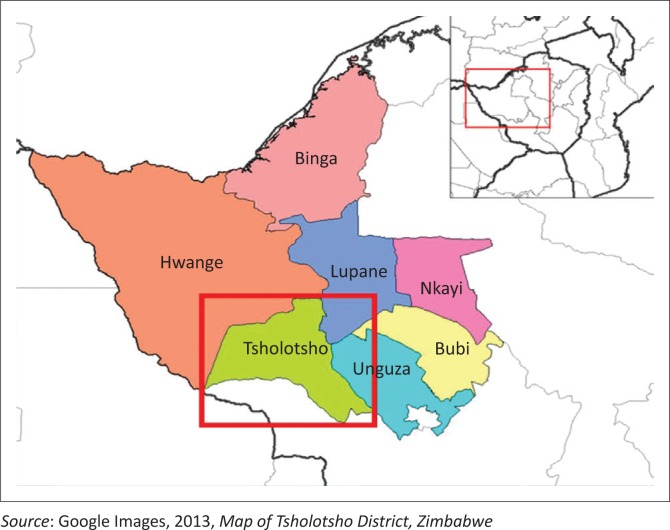
The map of Zimbabwe showing the location of Tsholotsho district in Matabeleland North province.

Tsholotsho is one of seven districts in the Matabeleland North province of Zimbabwe, with an estimated population of 115 119 people (Zimstat 2012). The district shares its borders with Bulilima, Lupane, Umguza and Hwange districts. Flooding has been a recurrent feature in Wards 5 and 8 in the district, with homes located along the Gwayi, Zombani and Manzamnyama rivers, and low-lying areas are regularly affected. These two wards are the most prone to flooding in the district because some communities are settled and undertake activities near the rivers. In map of Zimbabwe, the location of Tsholotsho district in Matabeleland North province is shown (Figure 1).

This study used a qualitative approach, whereby it sought to learn and understand the contribution of the indigenous knowledge of human communities in their natural setting as it relates to reducing the risk of natural disasters. The study adopted a case study design through studying Tsholotsho district affected by floods, as it sought to understand the contribution of indigenous knowledge of local communities from the participants’ lived experiences. Purposive sampling was used and data were solicited through interviews from 40 research participants. Higginbottom (2004) observes that in purposive sampling, the most important guiding principle is maximum variation, whereby researchers seek to include people who represent the widest variety of perspectives possible within the range specified by their purpose. An interview guide with semi-structured questions was used to collect data from 30 members of the community and 10 disaster risk reduction practitioners who are members of the Tsholotsho District Civil Protection Unit. The disaster risk reduction practitioners were included in order to understand whether they have considered using indigenous knowledge in managing disasters. A participant observation method was used to observe structural measures that were put in place as a result of indigenous knowledge to mitigate the impacts of flood disasters. Using the thematic analysis, various themes were derived from the data as detailed in the next section.

## Findings and discussions

This section of the article presents and discusses the findings of the study in order to show the contribution of indigenous knowledge to disaster risk reduction activities. In the discussion, the findings are related to the research objectives and are also compared with results from previous studies.

### Flood indigenous knowledge systems available to the communities in Tsholotsho

Members of the community affected by floods in Tsholotsho were found to possess a high level of indigenous knowledge. Such knowledge helped them to be aware of their vulnerability and to decide what action to take before, during and after flooding. From interviews conducted to decide with members of the community and disaster risk practitioners, it was mentioned that communities relied on their indigenous knowledge to forecast the magnitude of rains for the season, as well as to respond to the flood disaster. Outlined in the following sections are the indigenous knowledge practices for flood prediction and response in Tsholotsho district, as learnt from the respondents.

#### Indigenous knowledge systems for flood prediction

According to the respondents, they studied cloud patterns, the behaviour of certain animal species and changes on certain types of trees to predict whether the intensity of the forthcoming rains would result in floods. It was learnt from the respondents that the presence of *amayezi amnyama* (dark clouds) and the continuous crying and unsettledness of *inkanku* (a rain-making bird) are symbolic of heavy rains to come. Such rains have a potential to result in flooding in the district.

A male villager aged 36 years, from Mahlosi line, Siphepha area, narrated the following:

‘During rainy season, when we observe the continuous crying of an unsettled *inkankhu*, we know that heavy rains with a potential for flooding are imminent. We then ready ourselves to move up to higher grounds should the rains start falling.’

*Ukuhluma kwezihlahla* (the growth of new tree leaves that marks the onset of a new season) was also said to be a sign that the rainy season was about to begin. Table 1 is a summary of the indigenous knowledge of disaster risk reduction for dealing with floods in Tsholotsho district.

**TABLE 1:** Disaster risk reduction indigenous knowledge for flood prediction.

Available indigenous knowledge	Its symbolism to local communities	Frequency	Percent
*Amayezi amnyama* (dark clouds)	Heavy rains anticipated	24	60
Unsettled behaviour of *inkanku* (rain-making bird)	Heavy rains anticipated	12	30
*Ukuhluma kwezihlahla* (growing of new leaves from trees)	Onset of new rainy season	04	10

**Totals**	**-**	**40**	**100**

An analysis of the available indigenous knowledge systems of disaster risk reduction in Tsholotsho district (Table 1) shows that many people are familiar with dark clouds (60%), the rain-making bird (30%) and the growing of new leaves on trees (10%) to determine if the rains might result in flooding. This is so because the communities have been relying on these interventions to take appropriate action after forecasting flooding events.

The above findings are in line with results from previous studies. According to a study by Chinlampianga (2011) in Mizoram, northeast India, when clouds are thick and black in colour and are arranged perpendicular to the orbit of the sun, it symbolises that rain is approaching. In Swaziland, the presence of specific bird species in trees is understood to signal the onset of the rainy season for the local people and floods can be predicted by how high birds build their nests from river surfaces (Domfeh 2007).

#### Indigenous knowledge systems for flood response

Apart from the pre-disaster risk reduction measures for predicting flooding, communities in Tsholotsho also employ measures for post-disaster recovery phase in response to flood disasters. Their post-disaster indigenous knowledge measures, as indicated by the respondents based on the case study research, included land zoning (20; 50%), relocating livestock shelters to higher ground (16: 40%) and using locally available resources (4; 10%) to deal with flood disasters. Land zoning entails identifying safe land in the locality where communities would be moved in the event of flooding. The respondents who indicated land zoning cited that soon after the floods subside, they would leave the ‘safer land’ and return to their original homes. According to some respondents, shelter for livestock such as goats and cattle in flood-prone areas were located on higher ground so that flood waters would not reach them. They indicated that during flooding, their livestock survived, whilst those of people who built shelters in low-lying areas perished.

The study found that the communities had used wooden poles to create *amazibuko* (temporary foot bridges) along flooded rivers. These were especially meant for children whom villagers assisted to cross rivers when going to school. The research findings agree with a study by Baumwoll (2008), that indigenous knowledge adds value to the field of disaster risk reduction. In another study of the Lepcha community of Sikkim in India, Jha and Jha (2011) concluded that indigenous knowledge can be a useful input in a community’s endeavour to face challenges posed by natural disasters.

### The benefit of indigenous knowledge systems to local communities

The local communities in Tsholotsho district benefited through their application of the indigenous knowledge systems in dealing with disasters in a number of ways. According to 15 out of 30 (50%) respondents who are members of the communities, indigenous knowledge acts as an early warning against impending disasters. Six out of 30 (20%) respondents stated that indigenous knowledge can reveal the level of people’s vulnerability to disasters. Another 6 out of 30 (20%) respondents were of the opinion that indigenous knowledge identified elements within the society most at risk, whilst three out of 30 (10%) felt that indigenous knowledge helps to determine the course of action to be taken during disaster situations. This implies that local communities possess critical knowledge and life skills they can rely on for their continued survival. As a form of early warning, indigenous knowledge affords communities a chance to prepare for disasters and put measures in place in advance.

By revealing the level of vulnerability in the communities, indigenous knowledge helps people to take appropriate action in line with the magnitude of the conditions. According to the respondents, the community most at risk to flooding in Tsholotsho district are those settled along the Gwayi, Zombani and Manzamnyama rivers and in low-lying areas. This local knowledge has helped the communities to take appropriate measures in anticipating flooding in the district; for instance, through relocation. The findings support the study by Mutasa (2015), who found that communities in Buhera and Chikomba districts in Zimbabwe relied on indigenous knowledge to predict rainfall patterns. Indigenous knowledge also determines the course of action to be taken by local communities in response to a natural disaster. For instance, the study observed that members of the community had constructed a temporary bridge using wooden poles so as to help children cross the flooded river when going to school. The findings corroborate a study by the Australian Red Cross (2010) in the Pacific, who found that the indigenous knowledge of communities can mean better, faster and safer ways to respond to disasters, using the help available from members of local communities.

Despite the fact that the indigenous knowledge of local communities is of the utmost relevance, 70% of the respondents were of the opinion that their knowledge was underrated by the disaster risk reduction practitioners, whilst 30% had no idea whether the practitioners valued their knowledge or not. The following section highlights whether or not disaster risk reduction practitioners can benefit from the indigenous knowledge of local communities.

### The benefit of indigenous knowledge of local communities for practitioners

This study found that apart from the local communities benefitting from their indigenous knowledge and wisdom, the disaster risk reduction practitioners can also benefit from communities’ indigenous knowledge. According to commentaries 7 out of 10 (70%) respondents who are disaster risk reduction practitioners, practitioners can be effective in managing disasters if they combine their knowledge with that of the local communities and if they use communities’ knowledge for planning. One respondent, a male aged 46 years, who is a member of the Civil Protection Unit in Tsholotsho district, had this to say:

‘Communities’ indigenous knowledge is so important that if we disaster risk reduction practitioners can properly integrate it with our knowledge, we would be more effective in disaster response and recovery.’

Furthermore, respondents were of the view that practitioners can use the knowledge of local communities for improving disaster response and improving community participation.

The respondents noted that the knowledge of local communities is not always present in disaster risk reduction practitioners who are familiar with modern science; hence, practitioners as outsiders were found to be lacking in indigenous knowledge. Their view was that fusing the two types of knowledge may be a panacea to effective disaster risk reduction. In a study carried out in the South Pacific Region, Fletcher et al. (2013) also found that there was keen interest amongst the local communities to integrate indigenous knowledge with modern scientific approaches. The integration of indigenous knowledge with practitioners’ knowledge is also supported by Barrios, Coutinho and Medeiros (2012), who found that developing trans-disciplinary knowledge requires crossing multiple disciplinary boundaries and engaging scientific and non-scientific sources or practices.

Practitioners, as narrated by 6 out of 10 (60%) disaster risk reduction practitioners, can also use the indigenous knowledge of local communities for planning purposes to anticipate disasters. This implies that practitioners can rely on indigenous knowledge and use it for forecasting and predicting the magnitude of disasters. For instance, through studying the weather patterns and the behaviour of animal species, practitioners can forecast and plan for proper preventive, mitigation and preparedness measures to be followed. Chinlampianga (2011) also argued that local people’s knowledge of the weather and climate could be integrated with local planning strategies in mitigation and adaptation activities. As such, disaster risk reduction practitioners in Tsholotsho can immensely benefit from the indigenous knowledge of communities.

Using indigenous knowledge of communities can also contribute to improved activities in all phases of the disaster risk reduction cycle – prevention, mitigation, preparedness, response and recovery. According to 8 out of 10 (80%) disaster risk reduction practitioners, practitioners can build on local capacities of communities, such as their knowledge, experience and skills in dealing with flood disasters. Such contributions by local communities through their indigenous knowledge would improve disaster action, thereby saving lives and the property of affected communities. These findings correspond with results from previous studies. Bendito and Barrios (2016) found that effectively integrating knowledge systems in disaster situations would lead to greater and more sustainable results, together with a more efficient use of financial resources.

Lastly, disaster risk reduction practitioners can benefit from indigenous knowledge through improved community participation. One of the respondents, a 35-year-old male member of the Civil Protection Unit, had this to say:

‘If indigenous knowledge of local communities was seriously considered by practitioners, community participation would be improved. Through their participation, communities would easily accept ownership of development projects, making the projects more meaningful to local communities and sustainable.’

These findings support Jha and Jha (2011), who stated that indigenous knowledge is a precious national resource that can facilitate the process of disaster prevention, mitigation, preparedness and response in cost-effective, participatory and sustainable ways.

## Conclusion

The study drew a number of important conclusions. One major conclusion is that the indigenous knowledge of local communities plays an important part in disaster risk reduction activities. The study also found that communities possess valuable capacities in the form of indigenous knowledge, which can empower them to deal with all kinds of hazards and disasters. The study further concluded that it is highly possible for disaster risk reduction practitioners to integrate modern scientific knowledge with indigenous knowledge so that disaster risk reduction interventions become more effective. Whilst on the one hand, communities lack modern scientific knowledge and technology, on the other hand, disaster risk reduction practitioners lack indigenous knowledge. As such, the study also found that both members of the community and disaster risk reduction practitioners can immensely benefit from indigenous knowledge if they consider this knowledge when dealing with disaster events. Therefore, the indigenous knowledge of local communities is an indispensable empowerment tool that can be used in all stages of disaster risk reduction.

## Recommendations

This study recommends that local communities should consider practising their indigenous knowledge in disaster risk reduction activities that include prevention, mitigation, preparedness, response and recovery. It is also part of the study’s recommendations that disaster risk reduction practitioners should consider integrating their knowledge with the knowledge of local communities. Lastly, the study recommends that the indigenous knowledge of local communities be documented so that its recognition is increased.
